# Invasive Non-*Aspergillus* Mold Infections in Transplant Recipients, United States, 2001–2006

**DOI:** 10.3201/eid1710.110087

**Published:** 2011-10

**Authors:** Benjamin J. Park, Peter G. Pappas, Kathleen A. Wannemuehler, Barbara D. Alexander, Elias J. Anaissie, David R. Andes, John W. Baddley, Janice M. Brown, Lisa M. Brumble, Alison G. Freifeld, Susan Hadley, Loreen Herwaldt, James I. Ito, Carol A. Kauffman, G. Marshall Lyon, Kieren A. Marr, Vicki A. Morrison, Genovefa Papanicolaou, Thomas F. Patterson, Trish M. Perl, Mindy G. Schuster, Randall Walker, John R. Wingard, Thomas J. Walsh, Dimitrios P. Kontoyiannis

**Affiliations:** Author affiliations: Centers for Disease Control and Prevention, Atlanta, Georgia, USA (B.J. Park, K.A. Wannemuehler);; University of Alabama at Birmingham Medical Center, Birmingham, Alabama, USA (P.G. Pappas, J.W. Baddley);; Duke University Medical Center, Durham, North Carolina, USA (B.D. Alexander);; University of Arkansas for Medical Sciences, Little Rock, Arkansas, USA (E.J. Anaissie);; University of Wisconsin–Madison, Madison, Wisconsin, USA (D.R. Andes);; Stanford University School of Medicine, Stanford, California, USA (J.M. Brown);; Mayo Clinic, Jacksonville, Florida, USA (L.M. Brumble);; University of Nebraska Medical Center, Omaha, Nebraska, USA (A.G. Freifeld);; Tufts Medical Center, Boston, Massachusetts, USA (S. Hadley);; University of Iowa Hospital, Iowa City, Iowa, USA (L. Herwaldt);; City of Hope National Medical Center, Duarte, California, USA (J.I. Ito);; University of Michigan, Ann Arbor, Michigan, USA (C.A. Kauffman);; Veterans Affairs Ann Arbor Healthcare System–Medicine, Ann Arbor (C.A. Kauffman);; Emory University School of Medicine, Atlanta (G.M. Lyon);; Johns Hopkins Medical Institutions, Baltimore, Maryland, USA (K.A. Marr, T.M. Perl);; Fred Hutchinson Cancer Research Center, Seattle, Washington, USA (K.A. Marr);; University of Minnesota, Minneapolis, Minnesota, USA (V.A. Morrison);; Veterans Affairs Medical Center, Minneapolis (V.A. Morrison);; Memorial Sloan Kettering Cancer Center, New York, New York, USA (G. Papanicolaou);; University of Texas Health Sciences Center, San Antonio, Texas, USA (T.F. Patterson);; South Texas Veterans Healthcare System, San Antonio (T.F. Patterson);; Hospital of the University of Pennsylvania, Philadelphia, Pennsylvania, USA (M.G. Schuster);; Mayo Clinic, Rochester, Minnesota, USA (R. Walker);; University of Florida, Gainesville, Florida, USA (J.R. Wingard);; National Institutes of Health, Bethesda, Maryland, USA (T.J. Walsh);; MD Anderson Cancer Center, Houston, Texas, USA (D.P. Kontoyiannis)

**Keywords:** Mucorales, Fusarium, Scedosporium, mucormycosis, non-Aspergillus, fungi, organ transplant, epidemiology, invasive mold infections, zygomycosis, fusariosis, surveillance, United States, research

## Medscape CME

Medscape, LLC is pleased to provide online continuing medical education (CME) for this journal article, allowing clinicians the opportunity to earn CME credit.

This activity has been planned and implemented in accordance with the Essential Areas and policies of the Accreditation Council for Continuing Medical Education through the joint sponsorship of Medscape, LLC and Emerging Infectious Diseases. Medscape, LLC is accredited by the ACCME to provide continuing medical education for physicians.

Medscape, LLC designates this Journal-based CME activity for a maximum of 1 *AMA PRA Category 1 Credit(s)*^TM^. Physicians should claim only the credit commensurate with the extent of their participation in the activity.

All other clinicians completing this activity will be issued a certificate of participation. To participate in this journal CME activity: (1) review the learning objectives and author disclosures; (2) study the education content; (3) take the post-test with a 70% minimum passing score and complete the evaluation at www.medscape.org/journal/eid; (4) view/print certificate.

**Release date: September 23, 2011; Expiration date: September 23, 2012**

## Learning Objectives

Upon completion of this activity, participants will be able to:

- Distinguish the type of fungal infection most common in the current case series
- Evaluate patient characteristics associated with fungal infection after transplant
- Assess other factors associated with fungal infection after transplant
- Analyze the epidemiology of mucormycosis in the current case series

## Editor

**Karen L. Foster,** Technical Writer/Editor, *Emerging Infectious Diseases. Disclosure: Karen L. Foster has disclosed no relevant financial relationships.*

## CME Author

**Charles P. Vega, MD**, Associate Professor; Residency Director, Department of Family Medicine, University of California, Irvine. *Disclosure: Charles P. Vega, MD, has disclosed no relevant financial relationships.*

## Authors

Disclosures: **Benjamin J. Park, MD; Kathleen A. Wannemuehler, MSc; Janice M. Brown, MD; Lisa M. Brumble, MD; G. Marshall Lyon, MD, MMSc; Randall Walker, MD; Thomas J. Walsh, MD;** and **Dimitrios P. Kontoyiannis, MD,** have disclosed no relevant financial relationships. **Peter G. Pappas, MD,** has disclosed the following relevant financial relationships: served as an advisor or consultant for Pfizer Inc., Merck & Co., Inc., Astellas Pharma, Inc.; received grants for clinical research from Pfizer Inc., Merck & Co., Inc., Astellas Pharma, Inc. **Barbara D. Alexander, MD,** has disclosed the following relevant financial relationships: served as an advisor or consultant for bioMerieux, Bristol-Myers Squibb Company, Becton-Dickinson; received grants for clinical research from Pfizer Inc., Astellas Pharma, Inc., Charles River Laboratories. **Elias J. Anaissie, MD,** has disclosed the following relevant financial relationships: received grants for clinical research from Astellas Pharma, Inc., Millenium, Pfizer Inc. **David R. Andes, MD,** has disclosed the following relevant financial relationships: served as an advisor or consultant for Pfizer Inc., Merck & Co., Inc., Astellas; received grants for clinical research from Merck, Astellas Pharma, Inc. **John W. Baddley, MD, MSPH,** has disclosed the following relevant financial relationships: served as an advisor or consultant for Pfizer Inc., Merck & Co., Inc., Abbott; received grants for clinical research from Pfizer Inc. **Alison G. Freifeld, MD,** has disclosed the following relevant financial relationships: received grants for clinical research from Streck Inc., Chimerix. **Susan Hadley, MD,** has disclosed the following relevant financial relationships: served as an advisor or consultant for Merck & Co., Inc.; DSMB member. **Loreen Herwaldt, MD,** has disclosed the following relevant financial relationships: received grants for clinical research from 3M. **James I. Ito, MD,** has disclosed the following relevant financial relationships: served as an advisor or consultant for Sigma Tau; served as a speaker or a member of a speakers bureau for Astellas Pharma, Inc., Merck & Co., Inc., Pfizer Inc. **Carol A. Kauffman, MD,** has disclosed the following relevant financial relationships: received grants for clinical research from Merck & Co., Inc.; chair for the Data Adjudication Committee for Phase IV anidulafungin trial (Pfizer Inc.). **Kieren A. Marr, MD, **has disclosed the following relevant financial relationships: served as an advisor or consultant for Pfizer Inc., Merck & Co., Inc., Astellas Pharma, Inc.; received grants for clinical research from Merck & Co., Inc., Astellas Pharma, Inc. **Vicki A. Morrison, MD, **has disclosed the following relevant financial relationships: served as an advisor or consultant for Amgen Inc., Celgene Corporation; served as a speaker or a member of a speakers bureau for Amgen Inc., Celgene Corporation, Genentech Inc., Pfizer Inc. Genovefa Papanicolaou, MD, has disclosed the following relevant financial relationships: served as an advisor or consultant for Chimerix Inc., Merck & Co., Inc. **Thomas F. Patterson, MD,** has disclosed the following relevant financial relationships: served as an advisor or consultant for Pfizer Inc., Merck & Co., Inc., Astellas Pharma, Inc., Basilea & Toyon; served as a speaker for Pfizer Inc.; received grants for clinical research from Pfizer Inc., Merck & Co., Inc., Astellas Pharma, Inc., Basilea, Schering-Plough Corporation. **Trish M. Perl, MD, **has disclosed the following relevant financial relationships: served as an advisor or consultant for Hospira, BioMerieux, Pfizer Inc.; received grants for clinical research from Merck & Co., Inc., Sage. **Mindy G. Schuster, MD,** has disclosed the following relevant financial relationships: received grants for clinical research from Merck & Co., Inc. **John R. Wingard, MD,** has disclosed the following relevant financial relationships: served as an advisor or consultant for Merck & Co., Inc.; served as a speaker or a member of a speakers bureau for Pfizer Inc.
