# Supplementary material for: Invasive Non-Aspergillus Mold Infections in Transplant Recipients, United States, 2001–2006
Source: Emerg Infect Dis. 2011 Oct;17(10):1855–64. doi: 10.3201/eid1710.110087 (PMC3311117; doi:10.3201/eid1710.110087)
Supplement: Related texts

# Research article: Invasive Non-*Aspergillus* Mold Infections in Transplant Recipients, United States, 2001–2006

**DOI:** 10.3201/eid1710.110087

**Authors:** Benjamin J. Park, Peter G. Pappas, Kathleen A. Wannemuehler, Barbara D. Alexander, Elias J. Anaissie, David R. Andes, John W. Baddley, Janice M. Brown, Lisa M. Brumble, Alison G. Freifeld, Susan Hadley, Loreen Herwaldt, James I. Ito, Carol A. Kauffman, G. Marshall Lyon, Kieren A. Marr, Vicki A. Morrison, Genovefa Papanicolaou, Thomas F. Patterson, Trish M. Perl, Mindy G. Schuster, Randall Walker, John R. Wingard, Thomas J. Walsh, Dimitrios P. Kontoyiannis

**Affiliations:** Author affiliations: Centers for Disease Control and Prevention, Atlanta, Georgia, USA (B.J. Park, K.A. Wannemuehler);; University of Alabama at Birmingham Medical Center, Birmingham, Alabama, USA (P.G. Pappas, J.W. Baddley);; Duke University Medical Center, Durham, North Carolina, USA (B.D. Alexander);; University of Arkansas for Medical Sciences, Little Rock, Arkansas, USA (E.J. Anaissie);; University of Wisconsin–Madison, Madison, Wisconsin, USA (D.R. Andes);; Stanford University School of Medicine, Stanford, California, USA (J.M. Brown);; Mayo Clinic, Jacksonville, Florida, USA (L.M. Brumble);; University of Nebraska Medical Center, Omaha, Nebraska, USA (A.G. Freifeld);; Tufts Medical Center, Boston, Massachusetts, USA (S. Hadley);; University of Iowa Hospital, Iowa City, Iowa, USA (L. Herwaldt);; City of Hope National Medical Center, Duarte, California, USA (J.I. Ito);; University of Michigan, Ann Arbor, Michigan, USA (C.A. Kauffman);; Veterans Affairs Ann Arbor Healthcare System–Medicine, Ann Arbor (C.A. Kauffman);; Emory University School of Medicine, Atlanta (G.M. Lyon);; Johns Hopkins Medical Institutions, Baltimore, Maryland, USA (K.A. Marr, T.M. Perl);; Fred Hutchinson Cancer Research Center, Seattle, Washington, USA (K.A. Marr);; University of Minnesota, Minneapolis, Minnesota, USA (V.A. Morrison);; Veterans Affairs Medical Center, Minneapolis (V.A. Morrison);; Memorial Sloan Kettering Cancer Center, New York, New York, USA (G. Papanicolaou);; University of Texas Health Sciences Center, San Antonio, Texas, USA (T.F. Patterson);; South Texas Veterans Healthcare System, San Antonio (T.F. Patterson);; Hospital of the University of Pennsylvania, Philadelphia, Pennsylvania, USA (M.G. Schuster);; Mayo Clinic, Rochester, Minnesota, USA (R. Walker);; University of Florida, Gainesville, Florida, USA (J.R. Wingard);; National Institutes of Health, Bethesda, Maryland, USA (T.J. Walsh);; MD Anderson Cancer Center, Houston, Texas, USA (D.P. Kontoyiannis)

## Abstract

Recent reports describe increasing incidence of non-*Aspergillus* mold infections in hematopoietic cell transplant (HCT) and solid organ transplant (SOT) recipients. To investigate the epidemiology of infections with Mucorales, *Fusarium* spp., and *Scedosporium* spp. molds, we analyzed data from the Transplant-Associated Infection Surveillance Network, 23 transplant centers that conducted prospective surveillance for invasive fungal infections during 2001–2006. We identified 169 infections (105 Mucorales, 37 *Fusarium* spp., and 27 *Scedosporium* spp.) in 169 patients; 124 (73.4%) were in HCT recipients, and 45 (26.6%) were in SOT recipients. The crude 90-day mortality rate was 56.6%. The 12-month mucormycosis cumulative incidence was 0.29% for HCT and 0.07% for SOT. Mucormycosis incidence among HCT recipients varied widely, from 0.08% to 0.69%, with higher incidence in cohorts receiving transplants during 2003 and 2004. Non-*Aspergillus* mold infections continue to be associated with high mortality rates. The incidence of mucormycosis in HCT recipients increased substantially during the surveillance period.

Invasive mold infections are a major source of illness and death in transplant recipients. Non-*Aspergillus* invasive infections are of particular concern because of the following factors: the difficulty in distinguishing them clinically from *Aspergillus* spp. infections and from each other; their progressive and aggressive course; and the intrinsic resistance of many of these fungi to several antimicrobial agents, including voriconazole. Recent reports have described an increase at some medical centers in non-*Aspergillus* mold infections, particularly mucormycosis (formerly zygomycosis), often in persons receiving antifungal agents that have activity against *Aspergillus* spp (*1**–**4*).

Most reports have come from single institutions and might not be broadly representative of general trends. A comprehensive look at the modern epidemiology of these infections, including trends at multiple sites, can increase understanding of their public health implications. The Transplant-Associated Infection Surveillance Network (TRANSNET), a multicenter network of 23 academic tertiary care medical centers in the United States, performed prospective surveillance for invasive fungal infections (IFIs) during 2001–2006 (*5**–**7*). We analyzed the uniquely comprehensive TRANSNET cohort for epidemiology, patient demographics, clinical features, and outcomes of infections caused by the most common non-*Aspergillus* invasive molds detected: those of the order Mucorales, *Fusarium* spp., and *Scedosporium* spp.

## Methods

Study personnel based at each TRANSNET center reviewed records of patients who had undergone solid organ transplantation (SOT) or hematopoietic cell transplantation (HCT). Each IFI was evaluated to determine whether it met the criteria for a proven or probable IFI as defined by the European Organization for Research and Treatment of Cancer and Mycoses Study Group (EORTC-MSG) (*8*). Trained personnel at each site completed a detailed case report form for each identified proven and probable IFI. Data included demographics, clinical characteristics, clinical involvement, underlying diseases and conditions, and use of antifungal drugs. Investigators collected clinical data on all HCT recipients in whom an IFI developed, regardless of transplant date. Surveillance began in March 2001 and concluded in March 2006.

Cultures and histopathologic specimens were processed at the participating hospitals. Species were identified by using routine methods at the local laboratories. Fungal isolates were forwarded to the University of Alabama at Birmingham Fungal Reference Laboratory (Birmingham, AL, USA) and the Mycotic Diseases Branch at the Centers for Disease Control and Prevention (Atlanta, GA, USA), where species identification was confirmed by using morphologic and DNA-based methods. Patients in this analysis had an IFI and 1) a culture or histopathologic specimen consistent with Mucorales mold or 2) a culture from a clinical specimen positive for *Fusarium* spp. or *Scedosporium* spp. We excluded patients with invasive mold infection for whom diagnostic evidence was inconclusive.

All detected cases, regardless of transplant date, were described (surveillance cohort). In addition, the subset of cases in persons receiving transplants during the surveillance period was included for determination of the incidence of these infections (incidence cohort). Denominator data collected on identified patients undergoing transplant at each site during the surveillance period included date and type of transplant and date of last follow-up and clinical status at that time (alive, had undergone retransplant, IFI, dead). These data were used to calculate transplant-associated 12-month cumulative incidence. Estimated cumulative incidence was based on time to first infection after transplant and accounted for the competing risks for death, relapse of underlying disease, and retransplant. For HCT recipients, overall and transplant-specific 12-month cumulative incidence was calculated for mucormycosis and for fusariosis and scedosporiosis combined. For SOT patients, overall and transplant-specific incidence wase estimated only for mucormycosis because of low numbers of scedosporiosis and fusariosis. We estimated cumulative incidence using the cmprisk risk package version 2.2-1 in R version 2.11.1 (www.r-project.org).

We also explored the change in incidence of mold infections over time among HCT recipients. Because case counts can vary due to increases or decreases in the size of the denominator, we measured the changing epidemiology by estimating the incidence, according to transplant date. To do this, we classified the denominator of all transplant patients into 9 sequential subcohorts according to the 4-month period when the transplant was performed, as described (*5**,**7*). Twelve-month cumulative incidence for each of the 9 time periods (i.e., subcohorts) was calculated for mucormycosis and for fusariosis and scedosporiosis combined.

In addition, we attempted to estimate whether the underlying risk for infection in the overall population was changing because of changes in the numbers of allogeneic HCTs. To do this, we calculated the total follow-up time (in person-days) for each transplant type and plotted it for each period. Analysis was limited to the 21 sites that contributed data consistently during May 2002–April 2005, the last period for which complete data were available.

## Results

### Clinical Description of Cases

Of the 1,208 cases of proven or probable IFI in SOT recipients (*7*) and the 983 cases in HCT recipients (*5*) in TRANSNET, we detected 169 cases of Mucorales, *Fusarium* spp., or *Scedosporium* spp. infection in 166 transplant recipients, making these molds the most frequently identified molds, after *Aspergillus* within this patient population. The Mucorales (105 patients) were the most common of these molds, followed by *Fusarium* spp. (37 patients), and *Scedosporium* spp. (27 patients). Most cases occurred among HCT recipients (124 [73.4%] patients), compared with 45 (26.6%) cases in SOT recipients (Table 1).

**Table 1:** Distribution of Mucorales, *Fusarium*, and *Scedosporium* organisms causing infection in HCT and SOT recipients, as detected in TRANSNET, United States, 2001–2006*

Organism	No. (%) patients		
	HCT	SOT	Total
Mucorales	77 (62.1)	28 (62.2)	105 (62.1)
*Rhizopus* spp.	39 (50.6)	16 (57.1)	55 (52.4)
*Mucor* spp.	12 (15.6)	7 (25.0)	19 (18.1)
*Rhizomucor* spp.	7 (9.1)	0	7 (6.7)
*Cunninghamella* spp.	5 (6.5)	4 (14.3)	9 (8.6)
*Lichtheimia* spp.†	3 (3.9)	0	3 (2.9)
*Apophysomyces* spp.	1 (1.3)	0	1 (1.0)
*Syncephalastrum* spp.	1 (1.3)	0	1 (1.0)
Unspecified	9 (11.7)	1 (3.6)	10 (9.5)
Genus *Fusarium*	31 (25.0)	6 (13.3)	37 (21.9)
* F. solani*	9 (29.0)	1 (16.7)	10 (27.0)
* F. oxysporum*	0	2 (33.3)	2 (5.4)
* F. proliferatum*	2 (6.5)	0	2 (5.4)
* F. verticilloides*	1 (3.2)	0	1 (2.7)
Unspecified	19 (61.3)	3 (50.0)	22 (59.5)
Genus *Scedosporium*	16 (12.9)	11 (24.4)	27 (16.0)
* S. apiospermum*	13 (81.3)	6 (54.5)	19 (70.4)
* S. prolificans*	3 (18.8)	5 (45.5)	8 (29.6)
Total	124 (73.4)	45 (26.6)	169 (100)

*HCT, hematopoietic cell transplant; SOT, solid organ transplant; TRANSNET, Transplant-Associated Infection Surveillance Network. †This genus was formerly *Absidia.*

Among the Mucorales, *Rhizopus* was the most common genus (55 [52.4%] of 105 cases), followed by *Mucor* (19 [18.1%]) and unspecified Mucorales (10 [9.5%]) (Table 1). *F. solani* (10 [27.0%] cases) was the most common *Fusarium* species; however, we detected a large number of unspecified *Fusarium* spp. (22 [59.5%]). *S. apiospermum* (19 [70.4%]) and *S. prolificans* (8 [29.6%]) were the most common *Scedosporium* species (27 cases).

### Site Distribution

Numbers of cases varied by hospital and by region (Table 2). The 3 northeastern hospitals collectively reported 12 (7.1%) cases of non-*Aspergillus* mold infections during the surveillance period; 7 were caused by Mucorales. The 11 hospitals in the South reported 80 (47.3%) cases, of which 46 were caused by Mucorales. In the Midwest, the 5 participating hospitals detected 51 (30.2%) cases, and the 3 western hospitals detected 26 (15.4%) cases. The total number of Mucorales, *Fusarium* spp., or *Scedosporium* spp. infections varied substantially by site, ranging from 0 cases (3 sites) to 31 cases (1 site [18.3% of total cases]). The total number of transplants at each site for which follow-up was conducted as part of TRANSNET also varied widely, ranging from 89 to 2,551 HCTs and from 239 to 2,111 SOTs (Table 2). The median number of cases at a site was 8 (interquartile range 2–11 cases).

**Table 2:** Regional and hospital variability of Mucorales, *Fusarium* spp., and *Scedosporium* spp. invasive infections, as reported in TRANSNET, United States, 2001–2006*

US region,† hospital code	No. transplant recipients			No. (%) cases			
	HCT	SOT		Mucorales, n = 105	*Fusarium* spp., n = 37	*Scedosporium* spp., n = 27	Total, N = 169
Northeast				7 (6.7)	3 (8.1)	2 (7.4)	12 (7.1)
A	608	1,532		5 (4.8)	1 (2.7)	1 (3.7)	7 (4.1)
B	245	377		1 (1.0)	2 (5.4)	1 (3.7)	4 (2.4)
C	1,107	NA		1 (1.0)	0	0	1 (0.6)
South				46 (43.8)	23 (62.2)	11 (40.7)	80 (47.3)
D	2,551	NA		18 (17.1)	12 (32.4)	1 (3.7)	31 (18.3)
E	646	549		6 (5.7)	3 (8.1)	6 (22.2)	15 (8.9)
F	363	NA		5 (4.8)	3 (8.1)	1 (3.7)	9 (5.3)
G	523	1,201		4 (3.8)	2 (5.4)	2 (7.4)	8 (4.7)
H	342	1,532		5 (4.8)	1 (2.7)	1 (3.7)	7 (4.1)
I	808	377		4 (3.8)	0	0	4 (2.4)
J	89	728		3 (2.9)	0	0	3 (1.8)
K	2,040	239		1 (1.0)	1 (2.7)	0	2 (1.2)
L	110	1,210		0	1 (2.7)	0	1 (0.6)
M	449	NA		0	0	0	0
N	511	NA		0	0	0	0
Midwest				35 (33.3)	9 (24.3)	7 (25.9)	51 (30.2)
O	970	2,111		8 (7.6)	5 (13.5)	4 (14.8)	17 (10.1)
P	1,028	1,391		8 (7.6)	2 (5.4)	0	10 (5.9)
Q	361	1,210		8 (7.6)	0	0	8 (4.7)
R	315	2,111		5 (4.8)	0	3 (11.1)	8 (4.7)
S	546	755		6 (5.7)	2 (5.4)	0	8 (4.7)
West				17 (16.2)	2 (5.4)	7 (25.9)	26 (15.4)
T	1,512	NA		9 (8.6)	2 (5.4)	4 (14.8)	15 (8.9)
U	854	NA		8 (7.6)	0	3 (11.1)	11 (6.5)
V	1,019	NA		0	0	0	0

*TRANSNET, Transplant-Associated Infection Surveillance Network; HCT, hematopoietic cell transplant; SOT, solid organ transplant; NA, not available because of nonparticipation in surveillance for this transplant type. †Northeast: Massachusetts, New York, Pennsylvania; South: Alabama, Arkansas, Florida, Georgia, Maryland, North Carolina, Tennessee, Texas; Midwest: Iowa, Michigan, Minnesota, Nebraska, Wisconsin; West: California, Washington.

### Patient Characteristics (Surveillance Cohort)

Of the 169 case-patients, 96 (56.8%) were male, and 141 (91.6%) were white (Table 3); median age was 49 years (range 36–57 years). Proven IFIs comprised 95 (56.2%) cases. Active diabetes at the time of infection, either glucocorticoid-associated or as a preexisting condition, was present among 46 (43.8%) mucormycosis patients, 9 (56.8%) fusariosis patients, and 7 (25.9%) scedosporiosis patients. The overall crude mortality rate at 90 days was 56.6% (90/169).

**Table 3:** Characteristics of HCT and SOT patients* with proven or probable invasive Mucorales, *Fusarium* spp., or *Scedosporium* spp. infection, as reported in TRANSNET, United States, 2001–2006†

Characteristic‡	Mucorales, no. (%), n = 105	*Fusarium* spp., no. (%), n = 37	*Scedosporium* spp., no. (%), n = 27	Total, no. (%), N = 169
Male sex	58 (56.9)	26 (70.3)	12 (44.4)	96 (57.8)
White race	86 (90.5)	34 (94.4)	21 (91.3)	141 (91.6)
Proven IFI	61 (58.1)	21 (56.8)	13 (48.1)	95 (56.2)
Diabetes mellitus at time of fungal infection	46 (43.8)	9 (56.8)	7 (25.9)	62 (36.7)
Prior IFI	23 (21.9)	8 (19.5)	10 (24.4)	41 (24.3)
Prior aspergillosis	17 (16.2)	1 (2.7)	4 (14.8)	22 (13.0)
Antemortem clinical involvement§				
Pulmonary only	52 (53.1)	14 (38.9)	16 (59.3)	82 (50.9)
Sinus only	12 (12.2)	2 (5.6)	1 (3.7)	15 (9.3)
Skin only	9 (9.2)	3 (8.3)	0	12 (7.5)
Disseminated	13 (13.3)	8 (22.2)	7 (25.9)	28 (17.4)
Rhinocerebral only	3 (3.1)	1 (2.8)	0	4 (2.5)
Other¶	3 (3.1)	6 (16.7)	2 (7.4)	11 (6.8)
Death at 90 d	57 (58.2)	18 (51.4)	15 (57.7)	90 (56.6)
HCT	77 (73.3)	31 (24.3)	16 (59.3)	124 (73.4)
Underlying condition				
ALL	7 (9.1)	1 (3.2)	2 (12.5)	10 (8.1)
AML	21 (27.3)	11 (35.5)	3 (18.8)	35 (28.2)
CML	7 (9.1)	2 (6.5)	4 (25)	13 (10.5)
HL	4 (5.2)	1 (3.2)	0	5 (4)
NHL	15 (19.5)	8 (25.8)	3 (18.8)	26 (21)
AA	3 (3.9)	1 (3.2)	0	4 (3.2)
MM	5 (6.5)	1 (3.2)	1 (6.3)	7 (5.6)
MDS	10 (13)	1 (3.2)	1 (6.3)	12 (9.7)
HCT graft donor				
Autologous	8 (10.4)	4 (12.9)	0	12 (9.7)
Allogeneic matched-related	34 (44.2)	13 (41.9)	7 (43.8)	54 (43.5)
Allogeneic mismatched-related	4 (5.2)	4 (12.9)	2 (12.5)	10 (8.1)
Allogeneic unrelated	30 (39)	10 (32.3)	7 (43.8)	47 (37.9)
Other characteristics				
Neutropenia#	39 (50.6)	17 (54.8)	4 (25)	60 (48.4)
Steroids at diagnosis	59 (76.6)	24 (77.4)	14 (87.5)	97 (78.2)
Myeloablative preconditioning	46 (59.7)	18 (58.1)	11 (68.8)	75 (60.5)
Acute GvHD	32 (41.6)	7 (22.6)	6 (37.5)	45 (36.3)
Chronic GvHD	29 (37.7)	14 (45.2)	7 (43.8)	50 (40.3)
Steroid treatment	58 (75.3)	19 (61.3)	13 (81.3)	90 (72.6)
GvHD grade 1 or 2	18 (23.4)	4 (12.9)	4 (25.0)	26 (21.0)
GvHD grade 3 or 4	35 (45.4)	14 (45.2)	5 (31.3)	54 (43.5)
SOT	28 (26.7)	6 (51.4)	11 (40.7)	45 (26.6)
Organ transplanted				
Heart	3 (10.7)	1 (16.7)	0	4 (8.9)
Lung	8 (28.6)	2 (33.3)	9 (81.8)	19 (42.2)
Kidney	9 (32.1)	1 (16.7)	2 (18.2)	12 (26.7)
Liver	9 (32.1)	2 (33.3)	0	11 (24.4)
Organ rejection within previous 30 d	13 (48.1)	3 (50)	8 (72.7)	24 (54.5)

*Includes 3 patients with 2 infections each; 2 patients had separate *Fusarium* and *Scedosporium* infections; 1 person had separate mucormycosis and scedosporiosis. †Denominators vary because of missing data. HCT, hematopoietic cell transplant; SOT, solid organ transplant; TRANSNET, Transplant-Associated Infection Surveillance Network; IFI, invasive fungal infection; ALL, acute lymphocytic leukemia; AML, acute myelogenous leukemia; CML, chronic myelogenous leukemia; HL, Hodgkin lymphoma; NHL, non-Hodgkin lymphoma; AA, aplastic anemia; MM, multiple myeloma; MDS, myelodysplastic syndrome; GvHD, graft-versus-host disease. ‡Median ages (interquartile ranges) are as follows: patients with Mucorales infection, 51 y (39–57 y); patients with *Fusarium* spp. infection, 45 y (32–59 y); patients with *Scedosporium* spp. infection, 46 y (38–59 y); and all patients, 49 y (36–57 y). §Antemortem organ involvement was classified according to recorded sites of infection; disseminated infection was defined as infection in noncontiguous organs. ¶Esophageal (1 *Scedosporium* spp.); heart (3 Mucorales); blood (6 *Fusarium* spp., 1 *Scedosporium* sp.). #Defined as an absolute neutrophil count <500/μL, within 60 d before development of invasive fungal infection.

The lower respiratory tract was the most commonly involved site for all mold illnesses (53.1% of mucormycosis, 38.9% of fusariosis, and 59.3% of scedosporiosis) (Table 3). Disseminated disease (17.4% of all cases) was also frequent (13.3% of mucormycosis, 22.2% of fusariosis, and 25.9% of scedosporiosis).

Of the 124 HCT recipients with 1 of these infections, the most common indication for HCT was acute myelogenous leukemia (35 [28.2%] cases), followed by non-Hodgkin lymphoma (26 [21.0%] cases) (Table 3). Most HCT recipients received a human leukocyte antigen (HLA)–matched, allogeneic graft from a related donor (54 [43.5%] patients), followed by HCT recipients with an unrelated donor (47 [37.9%] patients) (Table 3). In the 60 days before diagnosis of infection, neutropenia was reported in 4 (25.0%) patients with scedosporiosis, 39 (50.6%) with mucormycosis, and 17 (54.8%) with fusariosis. Acute graft-versus-host disease was present in 32 (41.6%) patients with mucormycosis, 7 (22.6%) with fusariosis, and 6 (37.5%) with scedosporiosis.

Of the 45 transplant patients with mold infections who had received an SOT, 19 (42.2%) had received a lung and 12 (26.7%) had received a kidney (Table 3). Twenty-four (54.5%) patients experienced organ rejection in the 30 days before infection occurred.

Many cases occurred early after transplant, but late-onset infection was also prevalent. Among HCT recipients, 67 (54.4%) infections occurred <6 months after transplant; 17 (13.8%) occurred >2 years after transplant (Figure 1). Among SOT recipients, 17 (37.8%) infections occurred within the first 6 months; 15 (33.3%) occurred >2 years after transplant. Median time to IFI for liver transplant recipients was 81 days, compared with 533 for non–liver SOT recipients (p = 0.035).

**Figure 1:** Months from transplant to development of invasive mucormycosis, fusariosis, or scedosporiosis among hematopoietic cell (HCT) and solid organ (SOT) transplant recipients as reported in the Transplant-Associated Infection Surveillance Network, United States, 2001–2006.

### Exposure to Antifungal Drugs

Voriconazole was the most frequently administered antifungal agent before infection in mucormycosis patients (47 [44.7%]) (Table 4). In contrast, fewer patients with fusariosis (27.0%) or scedosporiosis (11.1%) had received voriconazole (Table 4). Of the 47 patients with mucormycosis who received voriconazole, 28 (59.6%) were receiving voriconazole as prophylaxis, 15 (31.9%) were receiving it as empiric therapy, and 4 (8.7%) were receiving it as treatment for a suspected or other proven fungal infection.

**Table 4:** Antifungal medications received before and after diagnosis of invasive mold infection, TRANSNET data, United States, 2001–2006*

Antifungal drug	Mucorales, no. (%), n = 105	*Fusarium* spp., no. (%), n = 37	*Scedosporium* spp., no. (%), n = 27	Total, no. (%), N = 169
Received before diagnosis				
Amphotericin B†	7 (6.7)	5 (13.5)	3 (11.1)	15 (8.9)
Fluconazole	35 (33.3)	17 (45.9)	5 (18.5)	57 (33.7)
Itraconazole	13 (12.4)	4 (10.8)	8 (29.6)	25 (14.8)
Voriconazole	47 (44.8)	10 (27.0)	3 (11.1)	60 (35.5)
Caspofungin	18 (17.1)	3 (8.1)	3 (11.1)	24 (14.2)
Posaconazole	0	1 (2.7)	0	1 (0.6)
Received after diagnosis				
Amphotericin B†	82 (78.1)	19 (51.4)	9 (33.3)	110 (65.1)
Fluconazole	4 (3.8)	0	0	4 (2.4)
Itraconazole	10 (9.5)	2 (5.4)	8 (29.6)	20 (11.8)
Voriconazole	39 (37.1)	20 (54.1)	15 (55.6)	74 (43.8)
Caspofungin	26 (24.8)	12 (32.4)	11 (40.7)	49 (29.0)
Posaconazole	24 (22.9)	3 (8.1)	3 (11.1)	30 (17.8)

*TRANSNET, Transplant-Associated Infection Surveillance Network. Numbers do not total 100% because of concomitant receipt of multiple antifungal agents. †Any amphotericin B formulation.

More mucormycosis patients received an amphotericin B formulation after diagnosis (78.1%) than did fusariosis (51.4%) and scedosporiosis (33.3%) patients (Table 4). Patients with fusariosis (20 [54.1%] of 37) and scedosporiosis (15 [55.6%] of 27) most commonly received voriconazole after IFI diagnosis.

### Incidence Cohort

Follow-up data were available for 15,820 HCT recipients from 22 sites. Among these, 17% died, 13% experienced relapse, and 1% underwent retransplant in the first 12 months after transplant. Mucormycosis was diagnosed in 44 (0.3%) persons. Overall 12-month cumulative incidence for mucormycosis in HCT recipients was 0.29% (Figure 2). Recipients of an organ or cells from an allogeneic HLA-unrelated donor had the highest 12-month cumulative incidence of mucormycosis at 0.85%; allogeneic HLA-matched related donors had a cumulative incidence of 0.58%; and allogeneic HLA-mismatched related donors had a 12-month cumulative incidence of 0.25% (Figure 2).

**Figure 2:** Twelve-month cumulative incidence for invasive A) Mucorales infections and B) combined *Fusarium* and *Scedosporium* spp. infections among donor types of hematopoietic cell transplant recipients reported in the Transplant-Associated Infection Surveillance Network, United States 2001–2006. Infection data by transplant type: allogenic URD, 2,483 patients, 20 with Mucorales infection, 9 with *Fusarium* or *Scedosporium* spp. infection; allogenic MRD, 3,499 patients, 19 with Mucorales infection, 11 with *Fusarium* or *Scedosporium* spp. infection; allogenic MMR, 427 patients, 1 with Mucorales infection, 2 with *Fusarium* or *Scedosporium* spp. infection; autologous, 9,326 patients, 4 with Mucorales infection, 2 with *Fusarium* or *Scedosporium* spp. infection; overall, 15,820 patients, 44 with Mucorales infection, 24 with *Fusarium* or *Scedosporium* spp. infection. URD, unrelated donor; MRD, matched related donor; MMR, mismatched related donor.

Follow-up data were available for 16,457 SOT recipients from 15 sites. Of these, 6% died; 2% of kidney transplant recipients returned to chronic dialysis; and 2% underwent retransplant within 12 months after the transplant. In 12 (0.07%) persons, mucormycosis was diagnosed within the first 12 months after transplant. In SOT recipients, the overall 12-month cumulative incidence of mucormycosis was 0.07% (Figure 3). Patients receiving lung transplants and liver transplants had the highest incidence of mucormycosis (0.18% and 0.16%, respectively). Four *Scedosporium* spp. (3 lung SOTs, 1 kidney) and 2 *Fusarium* spp. (1 lung SOT, 1 liver) infections occurred within the first 12 months after transplant (data not shown).

**Figure 3:** Twelve-month cumulative incidence for invasive Mucorales infections among solid organ transplant recipients reported in the Transplant-Associated Infection Surveillance Network, United States, 2001–2006. A total of 16,457 transplant patients had 12 Mucorales infections. Infection data by transplant type: lung, 1,179 patients, 2 with Mucorales infection; liver, 4,361 patients, 7 with Mucorales infection; kidney, 8,494 patients, 3 with Mucorales infection; pancreas, 1,174 patients, 0 with Mucorales infection; heart, 1,159 patients, 0 with Mucorales infection; other, 21 patients, 0 with Mucorales infection; small bowel, 69 patients, 0 with Mucorales infection.

We estimated cumulative incidence for mucormycosis and for *Fusarium* spp. and *Scedosporium* spp. infections combined for the 9 subcohorts from May–August 2002 through January–April 2005 (Figure 4). Cohorts ranged from ≈1,200 to 1,400 transplant recipients each. Estimated cumulative incidence of *Fusarium* spp. and *Scedosporium* spp. infections in the 9 subcohorts did not increase over time. Incidence ranged from 0% during January–April 2004 and September–December 2004 to a high of 0.30% (4 infections) during May–August 2004.

**Figure 4:** Changes in 12-month cumulative incidence for invasive Mucorales infections, compared with *Fusarium* and *Scedosporium* infections, reported in the Transplant-Associated Infection Surveillance Network, United States 2001–2005. Changes in the underlying hematopoietic cell transplant population, by transplant type, is shown for comparison. URD, unrelated donor; MMR, mismatched related donor; MRD, matched related donor.

The incidence of mucormycosis in HCT recipients varied, with generally higher incidences in 2003 and 2004 than in 2002. Initially, the 12-month cumulative incidence for mucormycosis was 0.08% (1 infection) for the cohort receiving transplants during May–August 2002. The cumulative incidence was higher for all subsequent cohorts and peaked at 0.69% (9 infections) in the cohort receiving transplants during September–December 2004. In the subsequent cohort that received transplants during January–April 2005, incidence declined to 0.16% (2 infections). These 39 Mucorales infections occurred at 13 of the 21 sites, with these 13 sites contributing 1–7 infections each. The pattern was similar across the 9 subcohorts when we excluded the site with most cases (site D) and limited the analysis to allogeneic transplants only (data not shown). In addition, the total follow-up time of allogeneic HCTs did not appreciably increase (Figure 4). Mucormycosis incidence rates appeared to decrease for the cohorts receiving transplants during the first 4 months (January–April) of each year in the 2003–2005 surveillance period.

## Discussion

We describe the modern epidemiology of common non-*Aspergillus* invasive mold infections at multiple tertiary care sites throughout the United States. During the TRANSNET surveillance period, the estimated cumulative incidence of mucormycosis among HCT subcohorts receiving transplants in 2003 and 2004 was generally higher than in 2002. Public health officials and clinicians should be aware of these emerging mold infections, particularly in susceptible hosts, such as transplant recipients.

The worrisome possibility of an increase in mucormycosis incidence has gained attention recently, particularly because this disease has an extremely high case-fatality rate (*9*,*10*). Most supporting information about the emergence of this mold has come from compilations of case series (*11**,**12*), single-center studies (*13**–**15*), or registries (*16**,**17*). One national study in France used administrative data to demonstrate an increase over a 10-year period (*18*). In contrast, our analysis is based on a comprehensive database from a surveillance program that included a large number of centers with a broad geographic distribution. Furthermore, all cases were independently evaluated by using standardized case definitions (EORTC-MSG criteria [*8*]).

Reasons for higher incidence of mucormycosis during 2003 and 2004 are not clear from these data. It is certainly possible that these are natural variations or that changes in practices (e.g., mold-active prophylaxis or immunosuppressive agents) or changes in the at-risk populations could be contributory factors. Although we did not see an increase in the numbers of transplants from allogeneic donors performed at the participating sites during this period, we did not have data on other conditions that increase risk for mucormycosis, such as diabetes mellitus or iron overload conditions. The higher incidence during certain periods did not result from a single institution; nor was it likely to be attributable to surveillance artifact, in which improved detection methods falsely suggest a changing trend because other common non-*Aspergillus* mold infections did not also increase during this period.

Another issue that has been reported recently is the frequency of breakthrough mucormycosis in patients receiving voriconazole (*3**,**4**,**19**–**21*). In these reports, progressive mucormycosis developed in patients who were receiving voriconazole for prophylaxis or treatment for another disease, or these patients were at higher risk for mucormycosis than were control populations (*2*). In our study, voriconazole was the most frequently reported antifungal drug used before mucormycosis developed, although it was used in fewer than half of all patients. The frequency of voriconazole use before development of mucormycosis is striking, especially in the context of these case reports; however, the implication of the broader use of this antifungal drug to the emergence of this mold is far from clear. Experimental models have demonstrated increased virulence of Mucorales after exposure to voriconazole (*22*), but other reports have noted an increase of this mold before introduction of voriconazole (*14**,**23*). In a randomized trial that used voriconazole as a prophylactic agent, incidence of mucormycosis was not higher among the intervention group than in the group who received fluconazole (*24*), although patients meeting enrollment criteria may not have been at high risk for mucormycosis (*25*). The increase might not be evidence of a causal relationship between voriconazole and mucormycosis but might instead reflect a parallel increase related to a changing patient risk profile, including patients at higher risk or increased time at risk, mainly through improved posttransplant survival (*25*). We did find that 1 factor associated with death in mucormycosis patients on bivariate analysis was prior use of voriconazole (data not shown); this finding could indicate that persons receiving voriconazole are more complicated transplant patients and therefore at higher underlying baseline risk for mucormycosis. Because our study did not collect medication data on uninfected controls or on global antimicrobial drug use practices, we were not able to correlate broader voriconazole use to the higher incidence of mucormycosis in this cohort.

We found a high degree of site-to-site variability in the number of cases reported. One hospital contributed 31 cases, 18 of which were caused by Mucorales; this number was nearly double that of the hospital with the second-highest number. No cases were detected at 3 sites. However, the site with 31 cases was also 1 of the sites with the highest number of HCTs performed per year. Whether this variability is a function of environmental or climate conditions, the underlying transplant population at that site, medical practices (e.g., prophylaxis, diagnostic interventions), or a combination of these factors is not known.

The variability in the incidence of infections from Mucorales in HCT recipients over time was also intriguing, with the lowest incidence in the cohorts who received transplants during the first 4 months of the year. One recent report described a similar finding for aspergillosis among HCT recipients (*26*). Whether this pattern reflects an underlying seasonal risk for mucormycosis is not known. However, a seasonal pattern is feasible; these molds exist in the environment, and environmental and temperature conditions can greatly influence their growth or sporulation (*27*). Further study of the seasonal risk for rare mold infections such as these most likely necessitates a larger cohort than that from TRANSNET; large national databases, including those using administrative codes, despite their poor predictive values (*28*), might be promising for estimating trends for such diseases.

Our data corroborate other reports regarding the clinical features of these mold infections (*11**,**29**–**33*). We found that even though most cases occurred soon after transplant, a large number occurred >6 months after transplant. A similar pattern occurs in other mold infections, such as those caused by *Aspergillus* spp., indicating the need for clinicians to be vigilant about mold infections, particularly a substantial length of time after transplant. Furthermore, we found that the clinical features of these 3 common non-*Aspergillus* mold infections were similar (with only bloodstream involvement being associated more often with fusariosis, as would be expected) (*30*). Not surprisingly, the pulmonary system and sinuses were the most common sites of infection for all molds studied, similar to infections caused by *Aspergillus* spp. These data highlight the similarities in features among mold infections and illustrate the difficulties in differentiating these infections on the basis of clinical features or organ involvement. Because correct treatment depends on the proper isolate identification, these results reinforce the need for major improvements in diagnostic testing, including development of tissue-based molecular identification methods (*34**,**35*) to correctly and efficiently identify the causative organism.

Our study had a few limitations. Because we collected data only on proven and probable IFIs (and omitted possible infections), as defined by EORTC-MSG criteria (*8*), we are likely to have underestimated disease in these populations. In addition, we were not able to systematically determine which, if any, of these infections resulted from donor-derived infection of the allograft. However, routine posttransplant surveillance conducted by transplant clinical teams, in conjunction with the United Network for Organ Sharing, did not indicate any such clusters.

Our large prospective parallel assessment of the modern epidemiology of these emerging, yet uncommon, non-*Aspergillus* molds demonstrated a generally higher incidence in mucormycosis in 2003 and 2004 than in 2002 in HCT recipients. More comprehensive and long-term surveillance for these emerging rare molds might be warranted to track trends and assess the changing landscape of these infections.

## References

[1] Husain S, Alexander BD, Munoz P, Avery RK, Houston S, Pruett T, Opportunistic mycelial fungal infections in organ transplant recipients: emerging importance of non-*Aspergillus* mycelial fungi. Clin Infect Dis. 2003;37:221–9. 10.1086/37582212856215

[2] Kontoyiannis DP, Lionakis MS, Lewis RE, Chamilos G, Healy M, Perego C, Zygomycosis in a tertiary-care cancer center in the era of *Aspergillus*-active antifungal therapy: a case-control observational study of 27 recent cases [see comment]. J Infect Dis. 2005;191:1350–60. 10.1086/42878015776383

[3] Marty FM, Cosimi LA, Baden LR. Breakthrough zygomycosis after voriconazole treatment in recipients of hematopoietic stem-cell transplants [comment]. N Engl J Med. 2004;350:950–2. 10.1056/NEJM20040226350092314985500

[4] Trifilio SM, Bennett CL, Yarnold PR, McKoy JM, Parada J, Mehta J, Breakthrough zygomycosis after voriconazole administration among patients with hematologic malignancies who receive hematopoietic stem-cell transplants or intensive chemotherapy. Bone Marrow Transplant. 2007;39:425–9. 10.1038/sj.bmt.170561417310132

[5] Kontoyiannis DP, Marr KA, Park BJ, Alexander BD, Anaissie EJ, Walsh TJ, Prospective surveillance for invasive fungal infections in hematopoietic stem cell transplant recipients, 2001–2006: overview of the Transplant-Associated Infection Surveillance Network (TRANSNET) database. Clin Infect Dis. 2010;50:1091–100. 10.1086/65126320218877

[6] Morgan J, Wannemuehler KA, Marr KA, Hadley S, Kontoyiannis DP, Walsh TJ, Incidence of invasive aspergillosis following hematopoietic stem cell and solid organ transplant: interim results of a prospective multicenter surveillance program. Med Mycol. 2005;43(Suppl 1):S49–58. 10.1080/1369378040002011316110792

[7] Pappas PG, Alexander BD, Andes DR, Hadley S, Kauffman CA, Freifeld A, Invasive fungal infections among organ transplant recipients: results of the Transplant-Associated Infection Surveillance Network (TRANSNET). Clin Infect Dis. 2010;50:1101–11. 10.1086/65126220218876

[8] Ascioglu S, Rex JH, de Pauw B, Bennett JE, Bille J, Crokaert F, Defining opportunistic invasive fungal infections in immunocompromised patients with cancer and hematopoietic stem cell transplants: an international consensus. Clin Infect Dis. 2002;34:7–14. 10.1086/32333511731939

[9] Singh N, Aguado JM, Bonatti H, Forrest G, Gupta KL, Safdar N, Zygomycosis in solid organ transplant recipients: a prospective, matched case–control study to assess risks for disease and outcome. J Infect Dis. 2009;200:1002–11. 10.1086/60544519659439

[10] Sun HY, Forrest G, Gupta KL, Aguado JM, Lortholary O, Julia MB, Rhino-orbital-cerebral zygomycosis in solid organ transplant recipients. Transplantation. 2010;90:85–92. 10.1097/TP.0b013e3181dde8fc20626095

[11] Roden MM, Zaoutis TE, Buchanan WL, Knudsen TA, Sarkisova TA, Schaufele RL, Epidemiology and outcome of zygomycosis: a review of 929 reported cases. Clin Infect Dis. 2005;41:634–53. 10.1086/43257916080086

[12] Bethge WA, Schmalzing M, Stuhler G, Schumacher U, Kröber SM, Horger M, Mucormycoses in patients with hematologic malignancies: an emerging fungal infection. Haematologica. 2005;90(Suppl):ECR22.16266913

[13] Chamilos G, Luna M, Lewis RE, Bodey GP, Chemaly R, Tarrand JJ, Invasive fungal infections in patients with hematologic malignancies in a tertiary care cancer center: an autopsy study over a 15-year period (1989–2003). Haematologica. 2006;91:986–9.16757415

[14] Marr KA, Carter RA, Crippa F, Wald A, Corey L. Epidemiology and outcome of mould infections in hematopoietic stem cell transplant recipients. Clin Infect Dis. 2002;34:909–17. 10.1086/33920211880955

[15] Garcia-Vidal C, Upton A, Kirby KA, Marr KA. Epidemiology of invasive mold infections in allogeneic stem cell transplant recipients: biological risk factors for infection according to time after transplantation. Clin Infect Dis. 2008;47:1041–50. 10.1086/59196918781877PMC2668264

[16] Neofytos D, Horn D, Anaissie E, Steinbach W, Olyaei A, Fishman J, Epidemiology and outcome of invasive fungal infection in adult hematopoietic stem cell transplant recipients: analysis of Multicenter Prospective Antifungal Therapy (PATH) Alliance registry. Clin Infect Dis. 2009;48:265–73. 10.1086/59584619115967

[17] Pagano L, Valentini CG, Posteraro B, Girmenia C, Ossi C, Pan A, Zygomycosis in Italy: a survey of FIMUA-ECMM (Federazione Italiana di Micopatologia Umana ed Animale and European Confederation of Medical Mycology). J Chemother. 2009;21:322–9.1956735410.1179/joc.2009.21.3.322

[18] Bitar D, Van Cauteren D, Lanternier F, Dannaoui E, Che D, Dromer F, Increasing incidence of zygomycosis (mucormycosis), France, 1997–2006. Emerg Infect Dis. 2009;15:1395–401. 10.3201/eid1509.09033419788806PMC2819884

[19] Lionakis MS, Kontoyiannis DP. Sinus zygomycosis in a patient receiving voriconazole prophylaxis. Br J Haematol. 2005;129:2. 10.1111/j.1365-2141.2005.05384.x15801950

[20] Imhof A, Balajee SA, Fredricks DN, Englund JA, Marr KA. Breakthrough fungal infections in stem cell transplant recipients receiving voriconazole. Clin Infect Dis. 2004;39:743–6. 10.1086/42327415356792

[21] Siwek GT, Dodgson KJ, de Magalhaes-Silverman M, Bartelt LA, Kilborn SB, Hoth PL, Invasive zygomycosis in hematopoietic stem cell transplant recipients receiving voriconazole prophylaxis. Clin Infect Dis. 2004;39:584–7. 10.1086/42272315356827

[22] Lamaris GA, Ben-Ami R, Lewis RE, Chamilos G, Samonis G, Kontoyiannis DP. Increased virulence of Zygomycetes organisms following exposure to voriconazole: a study involving fly and murine models of zygomycosis. J Infect Dis. 2009;199:1399–406. 10.1086/59761519358672

[23] Kontoyiannis DP, Wessel VC, Bodey GP, Rolston KV. Zygomycosis in the 1990s in a tertiary-care cancer center. Clin Infect Dis. 2000;30:851–6. 10.1086/31380310852735

[24] Wingard JR, Carter SL, Walsh TJ, Kurtzberg J, Small TN, Baden LR, Randomized, double-blind trial of fluconazole versus voriconazole for prevention of invasive fungal infection after allogeneic hematopoietic cell transplantation. Blood. 2010;116:5111–8. 10.1182/blood-2010-02-26815120826719PMC3012532

[25] Pongas GN, Lewis RE, Samonis G, Kontoyiannis DP. Voriconazole-associated zygomycosis: a significant consequence of evolving antifungal prophylaxis and immunosuppression practices? Clin Microbiol Infect. 2009;15(Suppl 5):93–7. 10.1111/j.1469-0691.2009.02988.x19754766

[26] Panackal AA, Li H, Kontoyiannis DP, Mori M, Perego CA, Boeckh M, Geoclimatic influences on invasive aspergillosis after hematopoietic stem cell transplantation. Clin Infect Dis. 2010;50:1588–97. 10.1086/65276120450414PMC3024009

[27] Kontoyiannis DP, Chamilos G, Hassan SA, Lewis RE, Albert ND, Tarrand JJ. Increased culture recovery of Zygomycetes under physiologic temperature conditions. Am J Clin Pathol. 2007;127:208–12. 10.1309/7KU5XWURYM0151YN17210526

[28] Chang DC, Burwell LA, Lyon GM, Pappas PG, Chiller TM, Wannemuehler KA, Comparison of the use of administrative data and an active system for surveillance of invasive aspergillosis. Infect Control Hosp Epidemiol. 2008;29:25–30. 10.1086/52432418171183

[29] Campo M, Lewis RE, Kontoyiannis DP. Invasive fusariosis in patients with hematologic malignancies at a cancer center: 1998–2009. J Infect. 2010;60:331–7. 10.1016/j.jinf.2010.01.01020138081

[30] Boutati EI, Anaissie EJ. *Fusarium*, a significant emerging pathogen in patients with hematologic malignancy: ten years' experience at a cancer center and implications for management. Blood. 1997;90:999–1008.9242529

[31] Lamaris GA, Chamilos G, Lewis RE, Safdar A, Raad II, Kontoyiannis DP. *Scedosporium* infection in a tertiary care cancer center: a review of 25 cases from 1989–2006. Clin Infect Dis. 2006;43:1580–4. 10.1086/50957917109292

[32] Husain S, Munoz P, Forrest G, Alexander BD, Somani J, Brennan K, Infections due to *Scedosporium apiospermum* and *Scedosporium prolificans* in transplant recipients: clinical characteristics and impact of antifungal agent therapy on outcome. Clin Infect Dis. 2005;40:89–99. 10.1086/42644515614697

[33] Idigoras P, Perez-Trallero E, Pineiro L, Larruskain J, López-Lopategui MC, Rodríguez N, Disseminated infection and colonization by *Scedosporium prolificans*: a review of 18 cases, 1990–1999. Clin Infect Dis. 2001;32:E158–65. 10.1086/32052111340550

[34] Balajee SA, Sigler L, Brandt ME. DNA and the classical way: identification of medically important molds in the 21st century. Med Mycol. 2007;45:475–90. 10.1080/1369378070144942517710617

[35] Muñoz-Cadavid C, Rudd S, Zaki SR, Patel M, Moser SA, Brandt ME, Improving molecular detection of fungal DNA in formalin-fixed paraffin-embedded tissues: comparison of five tissue DNA extraction methods using panfungal PCR. J Clin Microbiol. 2010;48:2147–53. 10.1128/JCM.00459-1020392915PMC2884522

# Research article: Invasive Non-*Aspergillus* Mold Infections in Transplant Recipients, United States, 2001–2006

**DOI:** 10.3201/eid1710.110087

**Authors:** Benjamin J. Park, Peter G. Pappas, Kathleen A. Wannemuehler, Barbara D. Alexander, Elias J. Anaissie, David R. Andes, John W. Baddley, Janice M. Brown, Lisa M. Brumble, Alison G. Freifeld, Susan Hadley, Loreen Herwaldt, James I. Ito, Carol A. Kauffman, G. Marshall Lyon, Kieren A. Marr, Vicki A. Morrison, Genovefa Papanicolaou, Thomas F. Patterson, Trish M. Perl, Mindy G. Schuster, Randall Walker, John R. Wingard, Thomas J. Walsh, Dimitrios P. Kontoyiannis

**Affiliations:** Author affiliations: Centers for Disease Control and Prevention, Atlanta, Georgia, USA (B.J. Park, K.A. Wannemuehler);; University of Alabama at Birmingham Medical Center, Birmingham, Alabama, USA (P.G. Pappas, J.W. Baddley);; Duke University Medical Center, Durham, North Carolina, USA (B.D. Alexander);; University of Arkansas for Medical Sciences, Little Rock, Arkansas, USA (E.J. Anaissie);; University of Wisconsin–Madison, Madison, Wisconsin, USA (D.R. Andes);; Stanford University School of Medicine, Stanford, California, USA (J.M. Brown);; Mayo Clinic, Jacksonville, Florida, USA (L.M. Brumble);; University of Nebraska Medical Center, Omaha, Nebraska, USA (A.G. Freifeld);; Tufts Medical Center, Boston, Massachusetts, USA (S. Hadley);; University of Iowa Hospital, Iowa City, Iowa, USA (L. Herwaldt);; City of Hope National Medical Center, Duarte, California, USA (J.I. Ito);; University of Michigan, Ann Arbor, Michigan, USA (C.A. Kauffman);; Veterans Affairs Ann Arbor Healthcare System–Medicine, Ann Arbor (C.A. Kauffman);; Emory University School of Medicine, Atlanta (G.M. Lyon);; Johns Hopkins Medical Institutions, Baltimore, Maryland, USA (K.A. Marr, T.M. Perl);; Fred Hutchinson Cancer Research Center, Seattle, Washington, USA (K.A. Marr);; University of Minnesota, Minneapolis, Minnesota, USA (V.A. Morrison);; Veterans Affairs Medical Center, Minneapolis (V.A. Morrison);; Memorial Sloan Kettering Cancer Center, New York, New York, USA (G. Papanicolaou);; University of Texas Health Sciences Center, San Antonio, Texas, USA (T.F. Patterson);; South Texas Veterans Healthcare System, San Antonio (T.F. Patterson);; Hospital of the University of Pennsylvania, Philadelphia, Pennsylvania, USA (M.G. Schuster);; Mayo Clinic, Rochester, Minnesota, USA (R. Walker);; University of Florida, Gainesville, Florida, USA (J.R. Wingard);; National Institutes of Health, Bethesda, Maryland, USA (T.J. Walsh);; MD Anderson Cancer Center, Houston, Texas, USA (D.P. Kontoyiannis)

## Earning Medscape CME Credit

To obtain credit, you should first read the journal article. After reading the article, you should be able to answer the following, related, multiple-choice questions. To complete the questions (with a minimum 70% passing score) and earn continuing medical education (CME) credit, please go to www.medscape.org/journal/eid. Credit cannot be obtained for tests completed on paper, although you may use the worksheet below to keep a record of your answers. You must be a registered user on Medscape.org. If you are not registered on Medscape.org, please click on the New Users: Free Registration link on the left hand side of the website to register. Only one answer is correct for each question. Once you successfully answer all post-test questions you will be able to view and/or print your certificate. For questions regarding the content of this activity, contact the accredited provider, CME@medscape.net. For technical assistance, contact CME@webmd.net. American Medical Association’s Physician’s Recognition Award (AMA PRA) credits are accepted in the US as evidence of participation in CME activities. For further information on this award, please refer to http://www.ama-assn.org/ama/pub/category/2922.html. The AMA has determined that physicians not licensed in the US who participate in this CME activity are eligible for *AMA PRA Category 1 Credits*™. Through agreements that the AMA has made with agencies in some countries, AMA PRA credit may be acceptable as evidence of participation in CME activities. If you are not licensed in the US, please complete the questions online, print the certificate and present it to your national medical association for review.Article Title: Invasive Non-*Aspergillus* Mold Infections in Transplant Recipients, United States, 2001–2006

## CME Questions

1. You are preparing a 35-year-old woman for hematopoietic cell transplantation (HCT) from a related donor, and you are concerned with the possibility of a fungal infection following transplantation. Which of the following infections was most common among the current cohort of transplant recipients?A. ScedosporiosisB. MucormycosisC. Chromoblastomycosis D. Fusariosis2. Which of the following statements regarding patient characteristics of individuals with fungal infection in the current study is most accurate?A. All cases had proven invasive fungal infectionB. The overall 90-day mortality rate exceeded 50%C. Nearly all patients had neutropenia before fungal infectionD. The prevalence of mucormycosis was highest among recipients of HCT from allogenic human leukocyte antigen mismatched related donors3. What else should you consider regarding factors associated with fungal infection in the current study?A. Most mold infections occurred in the gastrointestinal tractB. Liver transplant was particularly associated with a higher risk for mucormycosisC. There were no cases of late-onset infectionD. Voriconazole failed to prevent many infections, especially mucormycosis4. Which of the following statements regarding mucormycosis in the current study is most accurate?A. Rhizopus was the most common genusB. Renal transplants were associated with the highest incidence of mucormycosisC. The annual incidence rates of mucormycosis remained stable during the study periodD. Mucormycosis was most common in the months from January to April

## Activity Evaluation

**Table:** 

1. The activity supported the learning objectives.				
Strongly Disagree				Strongly Agree
1	2	3	4	5
2. The material was organized clearly for learning to occur.				
Strongly Disagree				Strongly Agree
1	2	3	4	5
3. The content learned from this activity will impact my practice.				
Strongly Disagree				Strongly Agree
1	2	3	4	5
4. The activity was presented objectively and free of commercial bias.				
Strongly Disagree				Strongly Agree
1	2	3	4	5
